# Supplementary material for: Are older adults of Rohingya community (Forcibly Displaced Myanmar Nationals or FDMNs) in Bangladesh fearful of COVID-19? Findings from a cross-sectional study
Source: PLoS One. 2021 Jun 23;16(6):e0253648. doi: 10.1371/journal.pone.0253648 (PMC8221477; doi:10.1371/journal.pone.0253648)
Supplement: S2 File — (PDF) [file pone.0253648.s002.pdf]

ARCED FOUNDATION
Impact of COVID-19 on Older Adults in Bangladesh
Questionnaire

Household ID [ ][ ][ ][ ]

Date: Day [ ][ ] Month [ ][ ] Year [ ][ ]

Respondent's name: -------------------- Father's name: ----------------------

Village/Camp: ------------------------ [ ][ ] Union: ------------------------- [ ]

Upazila: -------------------------- [ ] Mobile: [ ][ ][ ][ ][ ][ ][ ][ ][ ][ ][ ]

A. Respondent's socio-demographic characteristics

Sl No	Questions	Answer	Code	Skip to
1.	How old are you now?	(Years)		
2.	Sex of respondent	Male<br>Female	1<br>2	
3.	What is your current marital status?	Married<br>Widow/Widower<br>Divorced<br>Separated<br>Unmarried	1<br>2<br>3<br>4<br>5	
4.	Living arrangement	Alone<br>Spouse<br>Son<br>Daughter<br>Others, specify:	1<br>2<br>3<br>4<br>-96	
5.	Dependant on family for living	Yes<br>No	1<br>0	
6.	Can read or write	Yes<br>No	1<br>0	
7.	What is your source of COVID-19 related information?<br>(Multiple responses)	Television<br>Radio<br>Mobile<br>Internet<br>Social media<br>None	1<br>2<br>3<br>4<br>5<br>0	
8.	Family size	(Number)		

B. Non-communicable chronic conditions

		Are you currently suffering from any chronic disease?<br>[ENUMERATOR: Ask for each of the disease separately]<br>Code:<br>1. Yes<br>2. No<br>3. Don't know	Are you taking medication for this?<br>Code:<br>1. Yes<br>2. No<br>3. Don't know
1.	Arthritis		
2.	Hypertension		
3.	Heart disease		
4.	Stroke		
5.	Raised total cholesterol		
6.	Diabetes		
7.	Chronic lung disease		
8.	Chronic kidney disease		
9.	Cancer		
10.	Other non-communicable disease		
11.	Cataract		
12.	Other visual problem		
13.	Auditory problem		
14.	Other non-communicable disease (please specify)		

C. COVID-19 related information

1.	How often you are concerned about COVID-19?	1. Hardly<br>2. Sometimes/often
2.	How often you are overwhelmed by COVID-19?	1. Hardly<br>2. Sometimes/often
3.	How often do you feel loneliness during this COVID-19?	1. Hardly<br>2. Sometimes/often
4.	Compared to the months before the outbreak began, how has the frequency of your communication with close friends and family changed?	1. Same as previous<br>2. Less than previous
5.	Did you faced any difficulty obtaining food during COVID-19	1. No difficult<br>2. Faced difficulty
6.	Did you faced any difficulty getting medicine during COVID-19	1. No difficult<br>2. Faced difficulty

7.	Did you faced any difficulty receiving routine medical care during COVID-19	1. No difficult<br>2. Faced difficulty
8.	Do you think older adults are at highest risk of COVID-19?	1. Yes<br>2. No

D. Fear of COVID-19 Scale

1.	I am most afraid of coronavirus-19.	1. Strongly disagree<br>2. Disagree<br>3. Neither agree nor disagree<br>4. Agree<br>5. Strongly agree
2.	It makes me uncomfortable to think about coronavirus-19.	1. Strongly disagree<br>2. Disagree<br>3. Neither agree nor disagree<br>4. Agree<br>5. Strongly agree
3.	My hands become clammy when I think about coronavirus-19.	1. Strongly disagree<br>2. Disagree<br>3. Neither agree nor disagree<br>4. Agree<br>5. Strongly agree
4.	I am afraid of losing my life because of coronavirus-19.	1. Strongly disagree<br>2. Disagree<br>3. Neither agree nor disagree<br>4. Agree<br>5. Strongly agree
5.	When watching news and stories about coronavirus-19 on social media, I become nervous or anxious.	1. Strongly disagree<br>2. Disagree<br>3. Neither agree nor disagree<br>4. Agree<br>5. Strongly agree
6.	I cannot sleep because I’m worrying about getting coronavirus-19.	1. Strongly disagree<br>2. Disagree<br>3. Neither agree nor disagree<br>4. Agree<br>5. Strongly agree
7.	My heart races or palpitates when I think about getting coronavirus-19.	1. Strongly disagree<br>2. Disagree<br>3. Neither agree nor disagree<br>4. Agree<br>5. Strongly agree

Thank You!
