# Supplementary material for: Are older adults of Rohingya community (Forcibly Displaced Myanmar Nationals or FDMNs) in Bangladesh fearful of COVID-19? Findings from a cross-sectional study
Source: PLoS One. 2021 Jun 23;16(6):e0253648. doi: 10.1371/journal.pone.0253648 (PMC8221477; doi:10.1371/journal.pone.0253648)
Supplement: S3 File — (PDF) [file pone.0253648.s003.pdf]

আর্কেড ফাউন্ডেশন

ইমপ্যাক্ট অফ কোভিড-১৯ অন ওল্ডার এ্যাডাল্টস ইন বাংলাদেশ

প্রশ্নমালা

খানার আইডিঃ ☐☐☐☐ তারিখ:

দিন		মাস		বছর	

উত্তরদাতার নাম: -------------------------- বাবার নাম: -----------------------

গ্রাম/ক্যাম্প: ------------------------- ☐☐ ইউনিয়ন: -------------------------- ☐

উপজেলা: -------------------------- ☐ মোবাইলঃ ☐☐☐☐☐☐☐☐☐☐☐

A. উত্তরদাতার আর্থ-সামাজিক অবস্থা

ক্র. নং	প্রশ্নাবলী	উত্তর	কোড	স্কীপ
1.	আপনার বর্তমান বয়স কত?	(বছর)		
2.	উত্তরদাতার লিঙ্গ:	পুরুষ<br>মহিলা	1<br>2	
3.	আপনার বর্তমান বৈবাহিক অবস্থা কি?	বিবাহিত<br>বিধবা, বিপত্নীক<br>বিবাহ বিচ্ছেদ<br>আলাদা<br>অবিবাহিত	1<br>2<br>3<br>4<br>5	
4.	থাকার ব্যবস্থা / কার সঙ্গে বসবাস করেন?	একা<br>স্ত্রী<br>পুত্র<br>কন্যা<br>অন্যান্য (উল্লেখ করুন):	1<br>2<br>3<br>4<br>-96	
5.	আপনি কে আপনার জীবনযাত্রা নির্বাহের জন্য আপনার পরিবারের উপর নির্ভরশীল?	হ্যাঁ<br>না	1<br>0	
6.	পড়তে বা লিখতে পারেন?	হ্যাঁ<br>না	1<br>0	
7.	কোভিড-১৯ এর তথ্য আপনি কোন মাধ্যম থেকে পান?<br>(একাধিক উত্তর প্রযোজ্য)	টিভি<br>রেডিও<br>মোবাইল<br>ইন্টারনেট<br>সামাজিক মাধ্যম<br>কোনটাই না	1<br>2<br>3<br>4<br>5<br>0	
8.	পরিবারের সদস্য সংখ্যা	(নম্বর)		

B. নন-কমিউনিকেবল ক্রোনিক অবস্থা

		১.আপনি বর্তমানে কোনও দীর্ঘস্থায়ী রোগে ভুগছেন?<br>[এনুমেরেটর: প্রতিটি রোগের জন্য আলাদাভাবে জিজ্ঞাসা করুন]<br>কোড:<br>১. হ্যাঁ<br>২. না<br>৩. জানি না	২. আপনি কি এর জন্য ওষুধ খাচ্ছেন?<br>কোড:<br>১. হ্যাঁ<br>২. না<br>৩. জানি না
1.	বাত / আর্থরাইটিস		
2.	উচ্চ রক্তচাপ		
3.	হৃদরোগ		
4.	স্ট্রোক		
5.	উচ্চ মোট কোলেস্টেরল		
6.	ডায়াবেটিস		
7.	দীর্ঘস্থায়ী ফুসফুসের রোগ		
8.	দীর্ঘস্থায়ী কিডনি রোগ		
9.	ক্যান্সার		
10.	অন্যান্য অসংক্রামক রোগ		
11.	ছানি		
12.	অন্যান্য দৃষ্টিসংক্রান্ত সমস্যা		
13.	শ্রাবণ সমস্যা		
14.	অন্যান্য অসংক্রামক রোগ (অনুগ্রহ করে উল্লেখ করুন)		

C. কোভিড-১৯ সম্পর্কিত তথ্য

1.	কোভিড-১৯ মহামারী সম্পর্কে আপনি কি উদ্বিগ্ন/ চিন্তিত?	১. তেমন নয়<br>২. কখনও কখনও/প্রায়ই
2.	কোভিড-১৯ মহামারী নিয়ে আপনি কি অস্বস্তি বোধ করছেন?	১. তেমন নয়<br>২. কখনও কখনও/প্রায়ই
3.	কোভিড-১৯ মহামারীতে আপনি কি একাকীত্ব বোধ করছেন?	১. তেমন নয়<br>২. কখনও কখনও/প্রায়ই
4.	প্রকোপ শুরুর আগের মাসগুলির তুলনায়, আপনার ঘনিষ্ঠ বন্ধুবান্ধব এবং পরিবারের সাথে আপনার যোগাযোগ কীভাবে পরিবর্তিত হয়েছে?	১. আগের মতই আছে<br>২. আগের থেকে কমে গেছে

5.	কোভিড-১৯ মহামারীতে আপনি কি খাবার সংগ্রহ করতে সমস্যায় পড়েছেন?	১. সমস্যা হয়নি<br>২. সমস্যা হয়ছে
6.	কোভিড-১৯ মহামারীতে আপনি কি ঔষধ সংগ্রহ করতে সমস্যায় পড়েছেন?	১. সমস্যা হয়নি<br>২. সমস্যা হয়ছে
7.	কোভিড-১৯ মহামারীতে আপনি কি আপনার নিয়মিত চিকিৎসা পেতে সমস্যায় পড়েছেন?	১. সমস্যা হয়নি<br>২. সমস্যা হয়ছে
8.	আপনি কি মনে করেন ওল্ডার এ্যাডাল্টসদের কোভিড-১৯ এ সংক্রামিত হওয়ার সম্ভাবনা সবথেকে বেশি?	১. হ্যাঁ<br>২. না

D. কোভিড-১৯ সম্পর্কিত ভয়ের স্কেল

1.	আমি করোনাভাইরাস-১৯ নিয়ে খুব ভীত	১. দৃঢ়ভাবে অসম্মত<br>২. অসম্মত<br>৩. অসম্মতও না সম্মতও না<br>৪. সম্মত<br>৫. দৃঢ়ভাবে সম্মত
2.	আমি করোনাভাইরাস-১৯ সম্পর্কে ভাবতে অস্বস্তি বোধ করি	১. দৃঢ়ভাবে অসম্মত<br>২. অসম্মত<br>৩. অসম্মতও না সম্মতও না<br>৪. সম্মত<br>৫. দৃঢ়ভাবে সম্মত
3.	করোনাভাইরাস-১৯ সম্পর্কে ভাবলে আমার হাত ঘেমে ওঠে	১. দৃঢ়ভাবে অসম্মত<br>২. অসম্মত<br>৩. অসম্মতও না সম্মতও না<br>৪. সম্মত<br>৫. দৃঢ়ভাবে সম্মত
4.	আমি করোনাভাইরাস-১৯ এর কারনে আমার জীবন হারানোর ভয় পাচ্ছি	১. দৃঢ়ভাবে অসম্মত<br>২. অসম্মত<br>৩. অসম্মতও না সম্মতও না<br>৪. সম্মত<br>৫. দৃঢ়ভাবে সম্মত
5.	সোশ্যাল মিডিয়াতে করোনাভাইরাস-১৯ সম্পর্কিত খবর বা গল্পগুলো দেখে আমি উদ্বিগ্ন হয়ে পরি	১. দৃঢ়ভাবে অসম্মত<br>২. অসম্মত<br>৩. অসম্মতও না সম্মতও না<br>৪. সম্মত<br>৫. দৃঢ়ভাবে সম্মত
6.	করোনাভাইরাস-১৯ এ আক্রান্ত হওয়ার ভয়ে আমি ঘুমাতে পারিনা	১. দৃঢ়ভাবে অসম্মত<br>২. অসম্মত<br>৩. অসম্মতও না সম্মতও না<br>৪. সম্মত

		৫. দৃঢ়ভাবে সম্মত
7.	করোনাভাইরাস-১৯ এ আক্রান্ত হওয়ার ভয়ে আমার বুক ধরফর করে	১. দৃঢ়ভাবে অসম্মত<br>২. অসম্মত<br>৩. অসম্মতও না সম্মতও না<br>৪. সম্মত<br>৫. দৃঢ়ভাবে সম্মত

ধন্যবাদ!
