# Supplementary material for: Are older adults of Rohingya community (Forcibly Displaced Myanmar Nationals or FDMNs) in Bangladesh fearful of COVID-19? Findings from a cross-sectional study
Source: PLoS One. 2021 Jun 23;16(6):e0253648. doi: 10.1371/journal.pone.0253648 (PMC8221477; doi:10.1371/journal.pone.0253648)
Supplement: Attachment — Submitted filename: PONE-D-21-00805_reviewer Due 6 Feb 21 reviewed final.pdf [file pone.0253648.s004.pdf]

PLOS ONE

Do older adults of Rohingya community (Forcibly Displaced Myanmar Nationals or FDMNs) in Bangladesh are fearful of COVID-19? findings from a cross-sectional study

--Manuscript Draft--

**Manuscript Number:**	PONE-D-21-00805
**Article Type:**	Research Article
**Full Title:**	Do older adults of Rohingya community (Forcibly Displaced Myanmar Nationals or FDMNs) in Bangladesh are fearful of COVID-19? findings from a cross-sectional study
**Short Title:**	Fear of COVID-19 among Rohingya FDMNs in Bangladesh
**Corresponding Author:**	Sabuj Kanti Mistry<br>ARCED Foundation<br>Dhaka, BANGLADESH
**Keywords:**	Fear; FCV-19S; Rohingya; Forcibly Displaced Myanmar Nationals; COVID-19
**Abstract:**	Aim : This study aimed to assess the fear of COVID-19 and its associates among older Rohingya (Forcibly Displaced Myanmar Nationals or FDMNs) in Bangladesh.<br><br>Method : We conducted a cross-sectional survey among 416 older FDMNs aged 60 years and above living in camps of Cox's Bazar, Bangladesh. A semi-structured questionnaire was used to collect information on participants' socio-demographic and lifestyle characteristics, pre-existing non-communicable chronic conditions, and COVID-19 related information. Level of fear was measured using the seven-item Fear of COVID-19 Scale (FCV-19S). A multiple linear regression examined the factors associated with fear.<br><br>Results : Among 416 participants aged 60 years or above, the mean fear score was 14.8. Participants who were concerned about COVID-19 (β : 0.63, 95% CI: -0.26 to 1.53) and overwhelmed by COVID-19 (β : 3.54, 95% CI: 2.54 to 4.55) were significantly more likely to be fearful of COVID-19. Other factors significantly associated with higher level of fear were lesser frequency of communication during COVID-19, difficulty in obtaining food during COVID-19, perception that older adults are at highest risk of COVID-19 and receiving COVID-19 related information from Radio/television and friends/family/neighbours.<br><br>Conclusions : Our study highlighted that currently there is very little fear of COVID-19 among the older Rohingya FDMNs. This is probably due to lower COVID-19 mortality rate in camps, lack of awareness of the severity of the disease and brutality the older Rohingya adults faced in recent past which outraged this fear. However, further qualitative research is advised to find out the reasons behind this.
**Order of Authors:**	Sabuj Kanti Mistry
	ARM Mehrab Ali
	Farhana Akther
	Prince Peprah
	Sompa Reza
	Shaidatonnisha Prova
	Uday Narayan Yadav
**Additional Information:**	
**Question**	**Response**
**Financial Disclosure**<br><br>Enter a financial disclosure statement that describes the sources of funding for the	The author(s) received no specific funding for this work.

Powered by Editorial Manager® and ProduXion Manager® from Aries Systems Corporation

work included in this submission. Review the submission guidelines for detailed requirements. View published research articles from *PLOS ONE* for specific examples.<br><br>This statement is required for submission and **will appear in the published article** if the submission is accepted. Please make sure it is accurate.<br><br>**Unfunded studies**<br>Enter: *The author(s) received no specific funding for this work.*<br><br>**Funded studies**<br>Enter a statement with the following details:<br>• Initials of the authors who received each award<br>• Grant numbers awarded to each author<br>• The full name of each funder<br>• URL of each funder website<br>• Did the sponsors or funders play any role in the study design, data collection and analysis, decision to publish, or preparation of the manuscript?<br>• **NO** - Include this sentence at the end of your statement: *The funders had no role in study design, data collection and analysis, decision to publish, or preparation of the manuscript.*<br>• **YES** - Specify the role(s) played.<br><br>* typeset	
**Competing Interests**<br><br>Use the instructions below to enter a competing interest statement for this submission. On behalf of all authors, disclose any competing interests that could be perceived to bias this work—acknowledging all financial support and any other relevant financial or non-financial competing interests.<br><br>This statement **will appear in the published article** if the submission is accepted. Please make sure it is accurate. View published research articles from *PLOS ONE* for specific examples.	The authors have declared that no competing interests exist.

Powered by Editorial Manager® and ProduXion Manager® from Aries Systems Corporation

**NO authors have competing interests**<br><br>Enter: *The authors have declared that no competing interests exist.*<br><br>**Authors with competing interests**<br><br>Enter competing interest details beginning with this statement:<br><br>*I have read the journal's policy and the authors of this manuscript have the following competing interests: [insert competing interests here]*<br><br>* typeset	
**Ethics Statement**<br><br>Enter an ethics statement for this submission. This statement is required if the study involved:<br><br>• Human participants<br>• Human specimens or tissue<br>• Vertebrate animals or cephalopods<br>• Vertebrate embryos or tissues<br>• Field research<br><br>Write "N/A" if the submission does not require an ethics statement.<br><br>General guidance is provided below. Consult the submission guidelines for detailed instructions. **Make sure that all information entered here is included in the Methods section of the manuscript.**	The study protocol was approved by the institutional review board of Institute of Health Economics, University of Dhaka, Bangladesh (Ref: IHE/2020/1037). Both written and oral informed consents was sought from the participants (thumb impressions from those who were not able to read and write) before administering the survey. Participation was voluntary, and participants did not receive any compensation.

Powered by Editorial Manager® and ProduXion Manager® from Aries Systems Corporation

**Format for specific study types**<br><br>**Human Subject Research (involving human participants and/or tissue)**<br>• Give the name of the institutional review board or ethics committee that approved the study<br>• Include the approval number and/or a statement indicating approval of this research<br>• Indicate the form of consent obtained (written/oral) or the reason that consent was not obtained (e.g. the data were analyzed anonymously)<br><br>**Animal Research (involving vertebrate animals, embryos or tissues)**<br>• Provide the name of the Institutional Animal Care and Use Committee (IACUC) or other relevant ethics board that reviewed the study protocol, and indicate whether they approved this research or granted a formal waiver of ethical approval<br>• Include an approval number if one was obtained<br>• If the study involved *non-human primates*, add *additional details* about animal welfare and steps taken to ameliorate suffering<br>• If anesthesia, euthanasia, or any kind of animal sacrifice is part of the study, include briefly which substances and/or methods were applied<br><br>**Field Research**<br><br>Include the following details if this study involves the collection of plant, animal, or other materials from a natural setting:<br>• Field permit number<br>• Name of the institution or relevant body that granted permission	
**Data Availability**<br><br>Authors are required to make all data underlying the findings described fully available, without restriction, and from the time of publication. PLOS allows rare exceptions to address legal and ethical concerns. See the PLOS Data Policy and FAQ for detailed information.	Yes - all data are fully available without restriction

Powered by Editorial Manager® and ProduXion Manager® from Aries Systems Corporation

A Data Availability Statement describing where the data can be found is required at submission. Your answers to this question constitute the Data Availability Statement and **will be published in the article**, if accepted.<br><br>**Important:** Stating 'data available on request from the author' is not sufficient. If your data are only available upon request, select 'No' for the first question and explain your exceptional situation in the text box.<br><br>Do the authors confirm that all data underlying the findings described in their manuscript are fully available without restriction?	
**Describe where the data may be found in full sentences. If you are copying our sample text, replace any instances of XXX with the appropriate details.**<br><br>• If the data are **held or will be held in a public repository**, include URLs, accession numbers or DOIs. If this information will only be available after acceptance, indicate this by ticking the box below. For example: *All XXX files are available from the XXX database (accession number(s) XXX, XXX.).*<br>• If the data are all contained **within the manuscript and/or Supporting Information files**, enter the following: *All relevant data are within the manuscript and its Supporting Information files.*<br>• If neither of these applies but you are able to provide **details of access elsewhere**, with or without limitations, please do so. For example:<br><br>*Data cannot be shared publicly because of [XXX]. Data are available from the XXX Institutional Data Access / Ethics Committee (contact via XXX) for researchers who meet the criteria for access to confidential data.*<br><br>*The data underlying the results presented in the study are available from (include the name of the third party*	All relevant data are within the manuscript and its Supporting Information files.

and contact information or URL).<br>• This text is appropriate if the data are owned by a third party and authors do not have permission to share the data.<br><br>* typeset	
Additional data availability information:	

Manuscript Click here to access/download;Manuscript;Main text_Fear.docx

Do older adults of Rohingya community (Forcibly Displaced Myanmar Nationals or FDMNs) in Bangladesh are fearful of COVID-19? findings from a cross-sectional study

Sabuj Kanti Mistry[1,2,3*], ARM Mehrab Ali[1,4], Farhana Akther[5], Prince Peprah[2], Sompa Reza[6], Shaidatonnisha Prova[6], and Uday Narayan Yadav[2]

1. ARCED Foundation, 13/1, Pallabi, Mirpur-12, Dhaka, Bangladesh

2. Centre for Primary Health Care and Equity, University of New South Wales, Sydney, Australia

3. BRAC James P Grant School of Public Health, BRAC University, 68 Shahid Tajuddin Ahmed Sharani, Mohakhali, Dhaka-1212, Bangladesh

4. Innovations for Poverty Action, USA

5. Mawlana Bhashani Science and Technology University, Tangail, Bangladesh

6. Society for Health Extension and Development (SHED), Cox's Bazar, Bangladesh

* Correspondence to: Sabuj Kanti Mistry, Centre for Primary Health Care and Equity, University of New South Wales, Sydney, Australia, Mobile: +61 406863358 E-mail: smitra411@gmail.com

Short title: Fear of COVID-19 among Rohingya FDMNs in Bangladesh

Abstract

Aim: This study aimed to assess the fear of COVID-19 and its associates among older Rohingya (Forcibly Displaced Myanmar Nationals or FDMNs) in Bangladesh.

Method: We conducted a cross-sectional survey among 416 older FDMNs aged 60 years and above living in camps of Cox's Bazar, Bangladesh. A semi-structured questionnaire was used to collect information on participants' socio-demographic and lifestyle characteristics, pre-existing non-communicable chronic conditions, and COVID-19 related information. Level of fear was measured using the seven-item Fear of COVID-19 Scale (FCV-19S). A multiple linear regression examined the factors associated with fear.

Results: Among 416 participants aged 60 years or above, the mean fear score was 14.8. Participants who were concerned about COVID-19 (β: 0.63, 95% CI: -0.26 to 1.53) and overwhelmed by COVID-19 (β: 3.54, 95% CI: 2.54 to 4.55) were significantly more likely to be fearful of COVID-19. Other factors significantly associated with higher level of fear were lesser frequency of communication during COVID-19, difficulty in obtaining food during COVID-19, perception that older adults are at highest risk of COVID-19 and receiving COVID-19 related information from Radio/television and friends/family/neighbours.

Conclusions: Our study highlighted that currently there is very little fear of COVID-19 among the older Rohingya FDMNs. This is probably due to lower COVID-19 mortality rate in camps, lack of awareness of the severity of the disease and brutality the older Rohingya adults faced in recent past which outraged this fear. However, further qualitative research is advised to find out the reasons behind this.

Keywords

Fear; FCV-19S; Rohingya; Forcibly Displaced Myanmar Nationals; COVID-19

Introduction

The COVID-19 pandemic is one of the greatest calamities related to public health issue since World War II. The disease continue to spread across the world, with nearly 37 million confirmed cases in 188 countries with more than one million deaths up to 8th October [1]. World Health Organization (WHO) declared the disease as a global health emergency on 30 January 2020 [2]. All countries are implementing public health measures such as mobility restrictions, lockdowns, compulsory mask wearing, among others to reduce the transmission of this highly infectious disease and thus to control the morbidity as well as mortality rate.

However, public health emergencies in times of epidemics and pandemics such as the COVID-19 pandemic may cause intense fear and anxiety particularly among the most at-risk populations including refugees and displaced population, particularly the old age group [3, 4]. This is because refugee camps are transitory settlements that accommodate displaced individuals and families who have fled their home countries as a result of political instability, war and natural disasters [5]. In many refugee camps including the Cox's Bazar refugee camps of Bangladesh where Rohingya refuges are settled, there are poor social and physical conditions such as overcrowding, poor sanitation and absence of basic amenities such as water [5].

Rohingya people (Forcibly Displace Myanmar Nationals or FDMN) are majorly Muslims minority groups who had been exiled from Rakhine State, Myanmar, for the fear of persecution and death. Estimates show that hundreds of thousands of Rohingya people fled during August 2017 due to extreme violence and more than half of the deported were children. They took shelter in Cox's Bazar, a South-Easter district of Bangladesh located about 280 kilometres away from Rakhine state of Myanmar (the place from where they displaced), is now also the world's largest camp for

displaced people [6, 7]. Now almost 860,000 FDMNs are living in the 34 camps in Cox's Bazar with 51% being children, 45% adult, 4% older persons and 1% persons with disability [8].

The refugee camps are the most densely populated area where an average population density is about 40,000 inhabitants per square kilometre, with some areas approaching 70,000 inhabitants [7]. As a result, there is a high risk of transmission of COVID-19 in the area [9]. As of 30th September 2020, a total of 4479 COVID-19 positive cases have been confirmed in the Cox's Bazar district, of which 252 are in the Rohingya camps and the numbers are rapidly increasing [2]. People of all age groups are at risk of getting infected by this virus but older adults and people with pre-existing conditions are at higher risk of infection by COVID[10]. The emerging evidence shows that mortality related to COVID-19 is higher (15%) among the older adults worldwide, where almost 74% of the total death occurred among those above 65 years of age [11].

The UN Refugee Agency (UNHCR) indicates there are more than 31,500 FDMNs aged 60 years or older in the camps [12]. Therefore, age being a critical variable posing a major risk for COVID-19 may create serious emotional disturbances, insecurity anxiety and depression among old, aged people in the camps. The effect of COVID-19 among the Rohingya older FDMNs might be devastating because of the prevalence of multiple health challenges in the refugee camp like lack of healthcare facilities and services, existing higher prevalence of infectious and non-communicable chronic diseases, and poor knowledge of hygiene and practices [13]. Again, a contagion disease outbreak like the COVID-19 creates fear that can cause people to sometimes worry about their own health and the health of the loved ones, financial situation, changes in sleep or eating patterns, difficulty in concentrating, worsening physical and mental health problems [10].

It is worth mentioning here that older people from the refugee camp in Rohingya community may have experiences of fear of military brutality which may exacerbate the situation of their mental/psychological condition mix with the fear of pandemic COVID-19. In this line, few media reports highlighted the COVID fear among the Rohingya community of Cox's Bazar. However, there has been none scientific studies that measured the COVID-19 fear among this vulnerable group of population. Therefore, the current study was conducted to access the COVID-19 related level of fear and its associates among FDMN older adults aged 60 years or above in Bangladesh.

Materials and Methods

Study design and participants

This was a cross sectional study carried out among 416 FDMNs older adults aged 60 years and above residing in Rohingya refugee camps situated in the Cox's Bazar district in the South-Eastern part of Bangladesh in October 2020.

The sample size of 460 was calculated with the following assumptions: (unknown) prevalence of COVID-19 related fear=50%, sampling error=5%, Confidence Interval=95% and non-response rate=20%. A total of 416 Rohingya older adults aged 60 years and above responded to the study from 457 who were approached (response rate 91%). There is a total of 34 Rohingya camps located in Cox's Bazar district, from which Camp 08E (SSID CXB-210), located at *Ukhia* sub-district was conveniently selected (Figure 1). In absence of the list of the older adults in Rohingya camps, a convenience sampling technique was employed to identify the eligible participants from the selected camp. The enumerators continued visiting the households starting from one side of the camp and stopped once desired sample size was achieved. In the absence of an eligible participant in the approached household, the enumerators moved to the next one and if the household had

more than one older adult all of them were interviewed. The inclusion criteria included aged ≥60 years and FDMN status. The exclusion criteria included adverse mental conditions (clinically proved schizophrenia, bipolar mood disorder, dementia/cognitive impairment), a hearing disability, or unable to communicate.

Figure 1: Geographical location of the study area

Measures

Outcome measure

COVID-19 related fear was the primary outcome, which was measured using the seven-item Fear of COVID-19 Scale (FCV-19S) developed and validated by Ahorus et al. among the general Iranian population [illegible]. Participants' agreement/disagreement with the seven items was assessed using a five-point Likert-scale (ranging from 1= "strongly disagree," 3= "neither agree nor disagree," and 5= "strongly agree"). Hence, the cumulative score ranged from 7 to 35, where the higher the scores, the greater the fear of COVID-19. Reliability or the internal consistency of the scale among Rohingya older adults was acceptable (Cronbach's α = 0.89).

Explanatory variables

Explanatory variables considered in this study were age (categorized as 60-69, 70-79, and ≥80 years), gender (male/female), marital status (currently married/widow), family size (≤4 and more than 4), literacy (Illiterate/literate), living arrangements (living with other family members/living alone), dependence on family for living (yes/no), memory or concentration problem (no problem/low memory or concentration), pre-existing non-communicable chronic conditions

(yes/no), source of COVID-19 related information (Radio/Television, health workers, and friends/family/neighbors), concerned about COVID-19 (hardly, sometimes/often), overwhelmed by COVID-19 (hardly, sometimes/often), frequency of communication during COVID-19 (less than previous/same as previous), difficulty obtaining food during COVID-19 (no difficulty/faced difficulty), difficulty getting medicine during COVID-19 (no difficulty/faced difficulty), difficulty receiving routine medical care during COVID-19 (no difficulty/faced difficulty) and perceived that older adults at highest risk of COVID-19 (yes/no).

Self-reported information on the presence of pre-existing non-communicable conditions such as arthritis, hypertension, heart diseases, stroke, hypercholesterolemia, diabetes, chronic respiratory diseases, chronic kidney disease and cancer was also collected. This information was verified with health records of the participants (where these were available) and/or with family members. We did not collect information on the income and occupation as the participants were all unemployed and dependent on aid.

Data collection tools and techniques

A pre-tested semi-structured questionnaire in Bengali language was used to collect the information through face to face interview. Data were electronically recorded in Survey CTO mobile app (https://www.surveycto.com/) by two surveyors, who were local residents of Cox's Bazar, fluent in Rohingya dialects, and had previous experience of administering health survey in electronic platform. The enumerators were trained extensively before the data collection through half-day Zoom meeting.

The English version of the questionnaire was first translated to Bengali language and then back translated to English by two researchers to ensure the contents' consistency. The Bengali version of the tool was piloted among a small sample (n=10) of Rohingya older adults from the selected camp to refine the language in the final version. The participants approved the tool designed by research team without any corrections or suggestions.

Statistical analysis

Mean (SD) and frequency (%) was used to depict participants' characteristics. Independent t-tests and ANOVA evaluated the mean differences in the FCV-19S score by participants' characteristics. A multiple linear regression model was performed where variable selection was based on the backward elimination with Akaike Information Criterion (AIC) approach. Adjusted beta-coefficient (β) and 95% confidence interval (95% CI) are reported. All analyses were performed using the statistical software package Stata (Version 14.0).

Ethical approval

The study protocol was approved by the institutional review board of Institute of Health Economics, University of Dhaka, Bangladesh (Ref: IHE/2020/1037). Both written and oral informed consents was sought from the participants (thumb impressions from those who were not able to read and write) before administering the survey. Participation was voluntary, and participants did not receive any compensation. Written approval was also sought from the Office of the Refugee Relief and Repatriation Commissioner (RRRC) prior to accessing the camps and conducting the survey.

Patient and public involvement

Patients and/or public were not involved in development of research question, study design, conducting study and result dissemination.

Results

Participants' characteristics

Table 1 describes the socio-demographic characteristics of participants. Of 416 studied participants, 74% were aged 60-69 years, about 60% were male, 93% were married and almost 60% of them were residing with a household of more than four members. Nearly all the participants (98%) were functionally illiterate. A large number (87%) of the participants were living with the family members but half of them (46%) were not depending on their families for a living. Participants received information related to COVID-19 from different sources including Radio/Television (42.8%), health workers (72%) and friends/family/ neighbours (64%).

Table 1: Sociodemographic characteristics of the participants (N=416)

Characteristics	n	%
Total	416	100.0
Age (year, %)		
60 - 69	308	74.0
70-79	83	20.0
>= 80	25	6.0
Sex		
Male	251	60.3
Female	165	39.7
Marital status		
Married	389	93.5
Widow	27	6.5
Family size		
0-4	167	40.1
>4	249	59.9

Literacy		
Illiterate	406	97.6
Literate	10	2.4
Living arrangement		
Living with other family members	362	87.0
Living alone	54	13.0
Dependent on family for living		
No	191	45.9
Yes	225	54.1
*Source of COVID-19 related information		
Radio/Television	178	42.8
Health worker	298	71.6
Friends/family/neighbour	267	64.2

Fear among the participants

Mean fear score was 14.8 among the participants which indicates low level of fear among the participants. Table 2 shows the bivariate analysis with participants' characteristics and fear score of COVID-19. Here we found that age, gender, family size, dependence on family for a living, having problem in memory did not play a role in being afraid of COVID-19. Marital status of the participants, living arrangement, source of COVID-19 information from family/ friends and feeling overwhelmed by COVID-19 had a significant impact on the score of fear ($P<0.001$).Those who live with family members had higher fear score than those who were living alone ($P<0.001$). On the other hand, knowing information from friends/ family/ neighbours was responsible ($P<0.001$) for increasing the fear score than those who learned it from health workers or other media.

Difficulty in obtaining food or in receiving routine medical care during COVID-19 was statistically significant ($P<0.001$) with mean fear score of 17.0 and 16.1 respectively. The respondents who

were suffering from pre-existing chronic conditions had low level of COVID-19 related fear compared with those who hadn't had any such conditions (*P*<0.001).

Table 2: Fear score and bivariate analysis (N=416)

Characteristics	COVID-19 fear score		
	Mean	SD	*P*
Overall	14.8	4.3	
Age (year, %)			
60 - 69	15.0	4.5	0.083
70-79	14.2	3.9	
>= 80	14.8	3.3	
Sex			
Male	14.7	4.2	0.356
Female	15.1	4.4	
Marital status			
Married	15.0	0.2	<0.001
Widow	11.6	0.4	
Family size			
0-4	14.6	4.0	0.298
>4	15.0	4.5	
Living arrangement			
Living with other family members	15.2	4.3	<0.001
Living alone	12.6	3.6	
Dependent on family for living			
No	14.7	4.2	0.545
Yes	14.9	4.4	
Memory or concentration problems			
No problem	14.9	0.2	0.242
Low memory or concentration	14.2	0.6	
Pre-existing non-communicable chronic conditions			
No	15.6	4.2	<0.001
Yes	12.9	4.0	
*Source of COVID-19 related information			
Radio/Television	15.3	3.4	0.076
Health worker	14.5	4.8	0.029
Friends/family/neighbour	16.4	4.0	<0.001
Concerned about COVID-19			
Hardly	14.4	3.2	0.006

Sometimes/often	15.6	5.6	
Overwhelmed by COVID-19			
Hardly	12.0	3.3	<0.001
Sometimes/often	16.0	4.3	
Frequency of communication during COVID-19			
Same as previous	15.1	5.6	0.206
Less than previous	14.6	3.1	
Difficulty obtaining food during COVID-19			
No difficulty	13.7	4.1	<0.001
Faced difficulty	17.0	4.1	
Difficulty getting medicine during COVID-19			
No difficulty	14.6	4.5	0.165
Faced difficulty	15.3	4.1	
Difficulty receiving routine medical care during COVID-19			
No difficulty	14.2	4.6	<0.001
Faced difficulty	16.1	3.7	
Perceived that older adults at highest risk of COVID-19			
No	13.4	4.4	<0.001
Yes	15.6	4.0	

Independent factors associated with fear score

The full model included the socio-demographic characteristics and COVID-19 related variables (Table 1) deemed to be associated with COVID-19 related fear. The final model based on the lowest AIC are presented in table 3. In the adjusted model, Radio/Television as source of COVID-19 related information, friends/family/neighbour as source of COVID-19 related information, overwhelmed by COVID-19, difficulty obtaining food during COVID-19 and frequency of communication during COVID-19 were significantly associated with COVID-19 related fear among study participants (Table 3).

COVID-19 related fear was 1.07 units higher among participants who received COVID-19 related information from Radio/Television (β: 1.07, 95% CI: 0.19 to 1.95) and 2.62 units higher among

those who received COVID-19 related information from friends/family/neighbour (β: 2.62, 95% CI: 1.44 to 3.79). Similarly, the fear scores were 3.54 units higher among those who were overwhelmed by COVID-19 (β: 3.54, 95% CI: 2.54 to 4.55). On the other hand, the fear scores were 4.41 units lower among participants who had less communication with others during COVID-19 than previous (β: -4.41, 95% CI: -5.69 to -3.13). Meanwhile, the fear scores were 2.16 units higher among participants who had difficulty in obtaining food during COVID-19 (β: 2.16, 95% CI: 1.08 to -3.23).

Table 3: Factors associated with fear among the participants (N=416)

Characteristics	*β*	*P*	95% CI
Radio/Television as source of COVID-19 related information			
No	*Ref*		
Yes	1.07	0.017	0.19, 1.95
Friends/family/neighbour as source of COVID-19 related information			
No	*Ref*		
Yes	2.62	<0.001	1.44, 3.79
Concerned about COVID-19			
Hardly	*Ref*		
Sometimes/often	0.63	0.166	-0.26, 1.53
Overwhelmed by COVID-19			
Hardly	*Ref*		
Sometimes/often	3.54	<0.001	2.54, 4.55
Frequency of communication during COVID-19			
Same as previous	*Ref*		
Less than previous	-4.41	<0.001	-5.69, -3.13
Difficulty obtaining food during COVID-19			
No difficulty	*Ref*		
Faced difficulty	2.16	<0.001	1.08, 3.23
Perceived that older adults at highest risk of COVID-19			
No	*Ref*		
Yes	1.26	0.064	-0.07, 2.59

Discussion

To our knowledge, this is the first study to assess the level of fear and its associates among Rohingya FDMNs older adults in Cox's Bazar refugee camp. This study revealed two major findings. First, there was a relatively low level of fear among participants. Secondly, fear was associated with some socio-demographic characteristics of the participants and COVID-19 related factors.

Our study provides evidence to contradict the general assumption that COVID-19 has created enormous fear among all population groups, especially among the aged population in the world [15-17]. Although our findings are somewhat different with previous studies, it is worth mentioning that the included studies were not only performed with refugees in camps, but also used different measures and scales. Fear remains one of the key causes of stress, anxiety and mental problems (WHO, 2020a). Thus, our participants not fearing COVID-19 can be considered a positive condition for their mental and psychological wellbeing. However, this finding is particularly interesting and needs some explanations considering evidence that older adults are more likely to be infected and die from COVID-19 [18-21]. There can be several reasons for less fear for COVID-19 among the older Rohingya participants, such as poor health literacy about this infectious disease, previous brutal experience back in Myanmar, low COVID-19 cases in camp , low exposure to media in the camps and so on.

The present study further noted the factors associated with the higher level of fear among the older Rohingya population. We found that the participants who frequently become overwhelmed by the lethality of COVID-19 were more fearful than those who were indifferent to it. This is expected as people often become anxious and stressful of a disease when they are overwhelmed by its deadly

effect, which in turn make them fearful of it. Evidence suggests that older adults are more vulnerable to COVID-19 related death and disabilities than their younger counterpart in part due to the presence of several co-morbidities [22]. Thus, knowing that presently there is no cure for COVID-19 and that they are more vulnerable of facing its lethal outcomes they might have been fearful of being infected with it [23, 24].

Interestingly, the findings revealed that the participants who had relatively lesser communication with others during the pandemic than before was less fearful than those who had no effect with communication. This is probably because frequent discussion of the severity of COVID-19, its global reach and the number of deaths and disabilities it is causing around them and globally with friends, family and other acquaintances can make a person more fearful of it [25]. Our findings also support this, as we found that the participants who were receiving COVID-19 related information from their family/friends/neighbours were more fearful than those who are not getting information from them. In line with this, the present research also reports that receiving COVID-19 information from popular media sources i.e., Radio/Television also makes the participants fearful of it. Again, the similar explanation applies as the participants are getting several information related to the lethality of the disease through Radio/Television making them fearful of it.

We also found that the participants who had difficulty in obtaining food during this pandemic were more fearful than those who had not difficulty. Like many other countries of the world earning members in many of the families lost their job in Bangladesh during the lockdown imposed due to COVID-19, particularly among the production workforce [26]. Also, crops and vegetables were

not harvested in time or couldn’t transport to the market in time which caused food shortage and hiked off the price [27]. Peoples earning are reducing day by day due to the disease outbreak on one side, on the other hand, the price of the foods are increasing playing a significant role making them fearful of deadly effect of the disease.

Moreover, we found that the participants who believed that older adults are at highest risk of COVID-19 were more fearful. The possible explanation could be that the participants who had a feeling that they are at highest risk of being infected by COVID-19 and infection with COVID could be very lethal may have greater level of fear. Moreover, participants with such feeling would have been very anxious of being infected with the diseases and remain fearful of it [28].

To the best of our knowledge, this is the first study carried out among Rohingya older adults providing an insight of fear amid this COVID-19 pandemic. However, our study has some limitations too. First, we prepared our sampling frame based on the available household-level information in our data repository; thus, selection bias is possible. Second, in the absence of pre-pandemic estimates, we cannot assert that the increased prevalence of fear noted in our study could be attributed to the COVID-19 pandemic.

Some limitations of the present study worth remarking despite its novelty and strength. Firstly, the tool used to measure the level of fear was not developed with refugees. Second, recall bias and social desirability could be potentially inevitable in this study as all items and measures used were self-reported. Moreover, the relationship between fear and socio-demographic and lifestyle factors can be complex however our analysis was based on a cross-sectional survey data, which may not

be able to establish causation links. Our data prohibit making any causal and directional conclusions since we could not eliminate the potential effect due to reverse causality. These and other limitations may affect the veracity of the findings that may limit the representation and generalizability of the findings.

Conclusion

Low fear of COVID-19 pandemic among the oldest refugees in the Rohingya camp suggests that they are less concerned about the devastating appearance of the current pandemic. This may be due to not apprehending the actual situation, illiteracy/lack of knowledge about this infectious disease, previous brutal experience, and so forth. Fearlessness, though good in a sense of not having mental pressure among the most vulnerable peoples, could exacerbate the situation in the camp all in a sudden as they won't take any precautionary steps to combat the virus infection. Dissemination of the actual information, withdrawn the restriction on accessing media/ internet, availability of the health care facilities including the test, facilities will improve knowledge among the older Rohingya in the refugee camp will prevent further deterioration of the condition due to this COVID-19. Further qualitative study should have to be conducted to depict the real picture or the reasons behind the fearless situation of the older Rohingya FDMNs.

Supplementary file

S1. Data file of the study

Acknowledgements

We acknowledge the role of Sadia Sumaia Chowdhury, Programme Manager, ARCED Foundation and Muntasir Alam, Research Assistant, ARCED Foundation for their support in data collection for the study.

Contributors

SKM, AMI and UNY conceived and contributed to the design of the study. SKM and AMI carried out the data collection and analysis. SKM, AMI, FA, PP, SR, and SP contributed in writing the first draft of the manuscript. UNY commented extensively and edited the draft of the manuscript to finalize it. All authors read and approved the final version of the manuscript.

Data sharing statement

All relevant data are within the manuscript and its Supporting Information files.

Funding

This research was not funded.

Competing interest

The authors declare that they have no conflict of interest to disclose.

References

1. WHO. WHO Coronavirus Disease (COVID-19) Dashboard 2020. Available from: https://covid19.who.int/.
2. WHO. Rohingya Crisis: Situation Report #25 2020 [22/10/2020]. Available from: https://reliefweb.int/sites/reliefweb.int/files/resources/who-cxb-situation-report-25.pdf.
3. Peprah P. Ageing out of place in COVID-19 pandemic era: How does the situation look like for older refugees in camps? Archives of gerontology and geriatrics. 2020;90:104149-. Epub 2020/06/20. doi: 10.1016/j.archger.2020.104149. PubMed PMID: 32593091.
4. Peprah P, Gyasi RM. Stigma and COVID-19 crisis: A wake-up call. n/a(n/a). doi: 10.1002/hpm.3065.
5. Raju E, Ayeb-Karlsson S. COVID-19: How do you self-isolate in a refugee camp? International Journal of Public Health. 2020;65(5):515-7. doi: 10.1007/s00038-020-01381-8.
6. Homaira N, Islam M, Haider N. COVID-19 in the Rohingya refugee camps of Bangladesh: challenges and mitigation strategies. Global Biosecurity. 2020;1(4).
7. Kamal A-H, Huda M, Dell C, Hossain S, Ahmed S. Translational strategies to control and prevent spread of COVID-19 in the Rohiynga refugee camps in Bangladesh. Global Biosecurity. 2020;1(4).
8. WFP. WFP Bangladesh: Rohingya Refugee Response | Situation Report #41 (August 2020). 2020.
9. Vision W. Rohingya Refugee Response COVID-19 Report: World Vision Bangladesh. 2020.
10. CDC. Coronavirus Disease 2019 (COVID-19): People with Certain Medical Conditions 2020 [22/10/2020]. Available from: https://www.cdc.gov/coronavirus/2019-ncov/need-extra-precautions/people-with-medical-conditions.html.
11. Worldometer. Age, Sex, Existing Conditions of COVID-19 Cases and Deaths. 2020.
12. UNHCR. Joint Government of Bangladesh - UNHCR Population Factsheet 2020 [22/10/2020]. Available from: file:///C:/Users/z3430797/Downloads/GoB%20UNHCR%20Population%20Factsheet%20_%20March%2015.pdf.
13. Islam MM, Nuzhath T. Health risks of Rohingya refugee population in Bangladesh: a call for global attention. Journal of global health. 2018;8(2):020309-. doi: 10.7189/jogh.08.020309. PubMed PMID: 30410735.
14. Ahorsu DK, Lin C-Y, Imani V, Saffari M, Griffiths MD, Pakpour AH. The fear of COVID-19 scale: development and initial validation. International journal of mental health and addiction. 2020.
15. Nguyen HT, Do BN, Pham KM, Kim GB, Dam HTB, Nguyen TT, et al. Fear of COVID-19 Scale-Associations of Its Scores with Health Literacy and Health-Related Behaviors among Medical Students. International journal of environmental research and public health. 2020;17(11). Epub 2020/06/18. doi: 10.3390/ijerph17114164. PubMed PMID: 32545240; PubMed Central PMCID: PMCPMC7311979.
16. Pang NTP, Kamu A, Hambali NLB, Mun HC, Kassim MA, Mohamed NH, et al. Malay Version of the Fear of COVID-19 Scale: Validity and Reliability. Int J Ment Health Addict. 2020:1-10. Epub 2020/08/25. doi: 10.1007/s11469-020-00355-4. PubMed PMID: 32837437; PubMed Central PMCID: PMCPMC7333973.
17. Martínez-Lorca M, Martínez-Lorca A, Criado-Álvarez JJ, Armesilla MDC, Latorre JM. The fear of COVID-19 scale: Validation in spanish university students. Psychiatry research.

2020;293:113350. Epub 2020/08/11. doi: 10.1016/j.psychres.2020.113350. PubMed PMID: 32777619; PubMed Central PMCID: PMCPMC7396130.
18. Wu Z, McGoogan JM. Characteristics of and important lessons from the coronavirus disease 2019 (COVID-19) outbreak in China: summary of a report of 72 314 cases from the Chinese Center for Disease Control and Prevention. Jama. 2020;323(13):1239-42.
19. Guan W-j, Ni Z-y, Hu Y, Liang W-h, Ou C-q, He J-x, et al. Clinical characteristics of coronavirus disease 2019 in China. New England journal of medicine. 2020;382(18):1708-20.
20. Khoury R, Karam G. Impact of COVID-19 on mental healthcare of older adults: insights from Lebanon (Middle East). International Psychogeriatrics. 2020:1-4.
21. Niu S, Tian S, Lou J, Kang X, Zhang L, Lian H, et al. Clinical characteristics of older patients infected with COVID-19: A descriptive study. Archives of Gerontology and Geriatrics. 2020:104058.
22. Yadav UN, Rayamajhee B, Mistry SK, Parsekar SS, Mishra SK. A syndemic perspective on the management of non-communicable diseases amid the COVID-19 pandemic in low-and middle-income countries. Frontiers in Public Health. 2020;8:508.
23. Powell T, Bellin E, Ehrlich AR. Older Adults and Covid-19: The Most Vulnerable, the Hardest Hit. Hastings Center Report. 2020;50(3):61-3.
24. Parlapani E, Holeva V, Nikopoulou VA, Sereslis K, Athanasiadou M, Godosidis A, et al. Intolerance of uncertainty and loneliness in older adults during the COVID-19 pandemic. Frontiers in Psychiatry. 2020;11:842.
25. Mertens G, Gerritsen L, Duijndam S, Salemink E, Engelhard IM. Fear of the coronavirus (COVID-19): Predictors in an online study conducted in March 2020. Journal of Anxiety Disorders. 2020:102258.
26. UCAnews. Covid-19, job cuts and misery for Bangladesh's garment workers 2020 [24/11/2020]. Available from: https://www.ucanews.com/news/covid-19-job-cuts-and-misery-for-bangladeshs-garment-workers/89805#.
27. Heifer International. In Bangladesh COVID-19 Puts Farmers and Food Systems in Dire Straights 2020 [24/11/2020]. Available from: https://www.heifer.org/blog/covid-19-wreaks-havoc-on-crops-and-markets-in-bagladesh.html.
28. Vahia IV, Jeste DV, Reynolds CF. Older Adults and the Mental Health Effects of COVID-19. JAMA. 2020.

Tables

Table 1: Sociodemographic characteristics of the participants (N=416)

Characteristics	n	%
Total	416	100.0
Age (year, %)		
60 - 69	308	74.0
70-79	83	20.0
>= 80	25	6.0
Gender		
Male	251	60.3
Female	165	39.7

Marital status		
Married	389	93.5
Single	27	6.5
Family size		
0-4	167	40.1
>4	249	59.9
Literacy		
Illiterate	406	97.6
Literate	10	2.4
Living arrangement		
Living with other family members	362	87.0
Living alone	54	13.0
Dependent on family for living		
No	191	45.9
Yes	225	54.1
*Source of COVID-19 related information		
Radio/Television	178	42.8
Health worker	298	71.6
Friends/family/neighbour	267	64.2

Table 2: Fear score and bivariate analysis (N=416)

Characteristics	COVID-19 fear score		
	Mean	SD	*P*
Overall	14.8	4.3	
Age (year, %)			
60 - 69	15.0	4.5	0.083
70-79	14.2	3.9	
>= 80	14.8	3.3	
Gender			
Male	14.7	4.2	0.356
Female	15.1	4.4	
Marital status			
Married	15.0	0.2	<0.001
Single	11.6	0.4	
Family size			
0-4	14.6	4.0	0.298
>4	15.0	4.5	
Living arrangement			
Living with other family members	15.2	4.3	<0.001
Living alone	12.6	3.6	
Dependent on family for living			
No	14.7	4.2	0.545
Yes	14.9	4.4	
Memory or concentration problems			
No problem	14.9	0.2	0.242
Low memory or concentration	14.2	0.6	
Pre-existing non-communicable chronic conditions			
No	15.6	4.2	<0.001
Yes	12.9	4.0	
*Source of COVID-19 related information			
Radio/Television	15.3	3.4	0.076
Health worker	14.5	4.8	0.029
Friends/family/neighbour	16.4	4.0	<0.001
Concerned about COVID-19			
Hardly	14.4	3.2	0.006
Sometimes/often	15.6	5.6	
Overwhelmed by COVID-19			
Hardly	12.0	3.3	<0.001
Sometimes/often	16.0	4.3	
Feeling of loneliness			

Hardly	14.7	4.5	0.268
Sometimes/often	15.3	3.6	
Frequency of communication during COVID-19			
Same as previous	15.1	5.6	0.206
Less than previous	14.6	3.1	
Difficulty obtaining food during COVID-19			
No difficulty	13.7	4.1	<0.001
Faced difficulty	17.0	4.1	
Difficulty getting medicine during COVID-19			
No difficulty	14.6	4.5	0.165
Faced difficulty	15.3	4.1	
Difficulty receiving routine medical care during COVID-19			
No difficulty	14.2	4.6	<0.001
Faced difficulty	16.1	3.7	
Perceived that older adults at highest risk of COVID-19			
No	13.4	4.4	<0.001
Yes	15.6	4.0	

Table 3: Factors associated with fear among the participants (N=416)

Characteristics	*β*	*P*	95% CI
Radio/Television as source of COVID-19 related information			
No	*Ref*		
Yes	1.07	0.017	0.19, 1.95
Friends/family/neighbour as source of COVID-19 related information			
No	*Ref*		
Yes	2.62	<0.001	1.44, 3.79
Concerned about COVID-19			
Hardly	*Ref*		
Sometimes/often	0.63	0.166	-0.26, 1.53
Overwhelmed by COVID-19			
Hardly	*Ref*		
Sometimes/often	3.54	<0.001	2.54, 4.55
Frequency of communication during COVID-19			
Same as previous	*Ref*		
Less than previous	-4.41	<0.001	-5.69, -3.13
Difficulty obtaining food during COVID-19			
No difficulty	*Ref*		
Faced difficulty	2.16	<0.001	1.08, 3.23
Perceived that older adults at highest risk of COVID-19			
No	*Ref*		
Yes	1.26	0.064	-0.07, 2.59

Figure

Click here to access/download;Figure;Map_tiff.tif

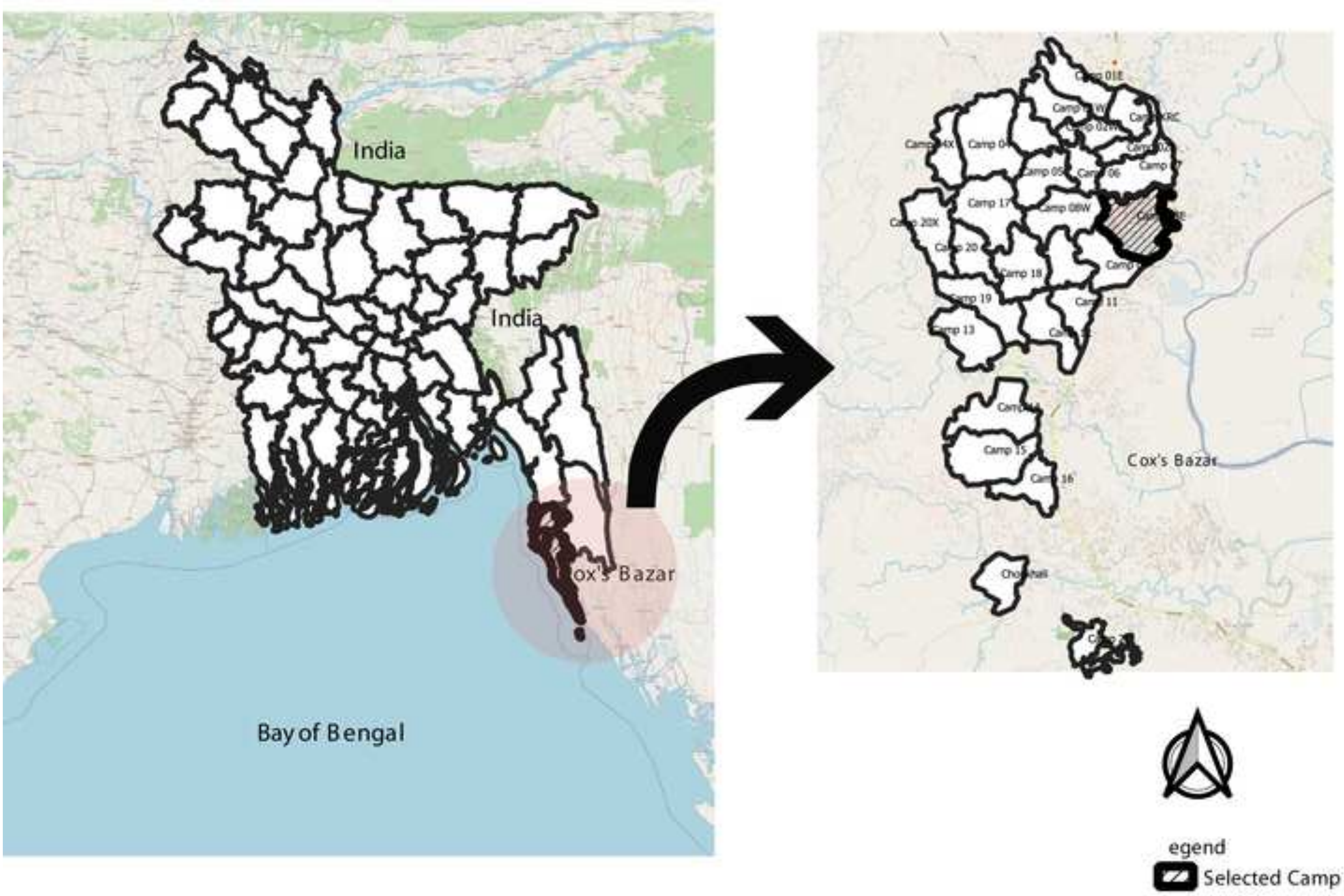

Data file

Click here to access/download
Supporting Information
S1.dta
